# Candidemia in the Neonatal Intensive Care Unit: A Retrospective, Observational Survey and Analysis of Literature Data

**DOI:** 10.1155/2017/7901763

**Published:** 2017-08-13

**Authors:** Giuseppina Caggiano, Grazia Lovero, Osvalda De Giglio, Giovanna Barbuti, Osvaldo Montagna, Nicola Laforgia, Maria Teresa Montagna

**Affiliations:** ^1^Department of Biomedical Science and Human Oncology, Hygiene Section, University of Bari “Aldo Moro”, Bari, Italy; ^2^Department of Biomedical Science and Human Oncology, General Pathology Section, University of Bari “Aldo Moro”, Bari, Italy; ^3^Neonatology and NICU Section, Azienda Ospedaliero-Universitaria Policlinico of Bari, Bari, Italy; ^4^Department of Biomedical Science and Human Oncology, Neonatology and NICU Section, University of Bari “Aldo Moro”, Bari, Italy

## Abstract

We evaluated the epidemiology of* Candida* bloodstream infections in the neonatal intensive care unit (NICU) of an Italian university hospital during a 9-year period as a means of quantifying the burden of infection and identifying emerging trends. Clinical data were searched for in the microbiological laboratory database. For comparative purposes, we performed a review of NICU candidemia. Forty-one candidemia cases were reviewed (overall incidence, 3.0 per 100 admissions).* Candida parapsilosis sensu stricto* (58.5%) and* C. albicans* (34.1%) were the most common species recovered. A variable drift through years was observed; in 2015, 75% of the cases were caused by non-*albicans* species. The duration of NICU hospitalization of patients with non-*albicans *was significantly longer than in those with* C. albicans* (median days, 10 versus 12). Patients with non-*albicans* species were more likely to have parenteral nutrition than those with* C. albicans* (96.3% versus 71.4%).* Candida albicans* was the dominant species in Europe and America (median, 55% and 60%; resp.); non-*albicans* species predominate in Asia (75%). Significant geographic variation is evident among cases of candidemia in different parts of the world, recognizing the importance of epidemiological data to facilitate the treatment.

## 1. Introduction

Although blood stream infection (BSI) due to* Candida *species (spp.) in the neonatal intensive care unit (NICU) is less frequent than that due to Gram-positive or Gram-negative bacteria, it has higher morbidity and mortality rates. In particular, among newborns with a birth weight < 1000 g, 4–8% will develop candidemia, which has a 30% mortality in this group of patients [1]. Newborns who survive frequently have long-term neurological impairment, including cerebral palsy, blindness, hearing impairment, cognitive deficits, and periventricular leukomalacia [2]. Risk factors for neonatal candidemia include prematurity, use of central venous lines, endotracheal tubes, parenteral nutrition, broad-spectrum antibiotic administration (especially third-generation cephalosporins), prolonged hospitalization, abdominal surgery, exposure to H2 blockers, and* Candida* colonization. Although* Candida albicans* is the most prevalent yeast pathogen, BSIs caused by* Candida* non-*albicans*, particularly* Candida parapsilosis* complex and* Candida glabrata* complex, have increased in recent years [2, 3].

This study aimed (i) to determine the epidemiology of* Candida* BSIs in the NICU of an Italian university hospital during 9 years of observation; (ii) to analyze the trend in species distribution; and (iii) to examine* in vitro* susceptibility to common antifungal drugs. Furthermore, for comparative purposes, a systematic review of studies concerning the distribution of* Candida* spp. causing candidemia in NICU patients is presented.

## 2. Materials and Methods

### 2.1. Study Design

A retrospective, observational survey of all consecutive cases of candidemia was conducted at the NICU (capacity of 8 beds; level III) of a university hospital in Southern Italy, from January 1, 2007, to December 31, 2015. The number of annual admissions ranged from 135 to 169, with no significant variation during the period of study. All of the neonates who had at least one positive blood culture for* Candida *spp. and signs or symptoms of infection were considered in this study. Only the first episode of candidemia was reported for patients with recurrent or subsequent episodes. Clinical data were searched for in the microbiological laboratory database and included sex, gestational age, birth weight, and predisposing risk factors for* Candida* BSI (i.e., intravascular devices, prolonged antibiotics, administration of total parental nutrition, and prolonged hospitalization).

### 2.2. Definitions

Extremely low birth weight (ELBW) infants were defined as those with a birth weight ≤ 1000 g, very low birth weight (VLBW) infants were those with a birth weight <1500 g, and low birth weight infants were those with a birth weight < 2500 g. Prolonged antibiotic use was defined as >14 days of continuous administration. Late-onset sepsis (LOS) was defined as infection occurring for >48 h of life. Candidemia was considered as probably catheter-related when semiquantitative culture of the catheter tip yielded >15 colony-forming units of* Candida*.

### 2.3. Laboratory Procedures

Blood cultures were performed using a lysis-centrifugation system (Isolator; DuPont Co., Wilmington, DE, USA). The samples were cultured on two plates of Sabouraud dextrose agar with 0.05% chloramphenicol (BioRad, Marnes-la-Coquette, France) and then incubated at 36°C (±1) and 28°C (±1). The samples were examined daily for 10 days. The isolates were identified using standard procedures (morphology on cornmeal agar plates, germ-tube production in serum, and ability to grow at 37°C and 42°C) and biochemical analysis using two methods, the Vitek 2 system and ID 32C panels (Bio-Merieux, Rome, Italy), to obtain accurate results. All strains were frozen at −70°C until further investigations [4].* Candida parapsilosis* complex genotyping was performed by PCR amplification as reported previously [5, 6].

Antifungal susceptibility tests to five antifungal drugs (anidulafungin, fluconazole, caspofungin, micafungin, and amphotericin B) were performed for all* Candida* spp., using the Sensititre YeastOne technique (SYO-09 panel; Trek Diagnostic Systems, Ltd., East Grinstead, England).

The susceptibility values were interpreted taking into account the species-specific clinical breakpoints (CBPs) suggested by the Clinical Laboratory Standards Institute (CLSI) subcommittee for the most common species of* Candida* [7]. The epidemiological cut-off values were used to define wild-type and non-wild-type isolates if no CBPs were available from the CLSI [8, 9]. Minimum inhibitory concentration (MIC) data are presented as MIC_50_ (MIC causing inhibition of 50% of isolates) and MIC_90_ (MIC causing inhibition of 90% of isolates).

### 2.4. Statistical Analysis

The Shapiro–Wilk test was used to test the normal distribution of data. Non-normally distributed data are expressed as median and interquartile range (IQR) and were compared using the Mann–Whitney* U* test. Categorical data are expressed as number and percentage and were compared using *χ*^2^ or Fisher's exact test. All *p* values are two-tailed, and statistical significance was defined as *p* < 0.05 (Social Sciences (SPSS) software 10 for Mac OS X; SPSS Inc., Chicago, IL, USA).

### 2.5. Literature Review

A review of full-text articles that were published in English from January 2000 to February 2015 was performed. The MEDLINE database was used for the bibliographic research, using the following key words: “neonatal candidemia”, “candidemia neonatal intensive care unit”, “*Candida *neonatal intensive care unit, and “NICU candidemia”. Additionally, the bibliographies of the selected articles were reviewed for relevant publications.

The exclusion criteria were as follows: articles that reported a period of study prior to 2000; letters, randomized, controlled trials; and studies that reported a total number of* Candida* BSIs less than five. The following data were collected from each selected study: geographic location, year of publication, study period, type of study, incidence, influencing factors candidemia, total number of isolated* Candida* spp., and relative proportion of each of the* Candida* spp.

## 3. Results

### 3.1. Analysis of Cases in the NICU

A total of 41 infants with* Candida* infection were reviewed. The overall incidence of candidemia was 3.0 per 100 NICU admissions (range, 2.2–3.0). The male : female ratio was 1.6 : 1. The cohort had a median gestational age of 30 weeks (29–31 weeks) and a median birth weight of 1110 g (900–1345 g). The majority of candidemia episodes occurred in VLBW infants (56.1%). The median duration of the total hospital stay was 11 days (8–14 days). Candidemia was catheter-related in 23 cases (56.1%). All* Candida* infections were classified as LOS. At the moment of candidemia, only ELBW infants were receiving antifungal prophylaxis with fluconazole (3 mg/kg/day).

**Figure 1:**
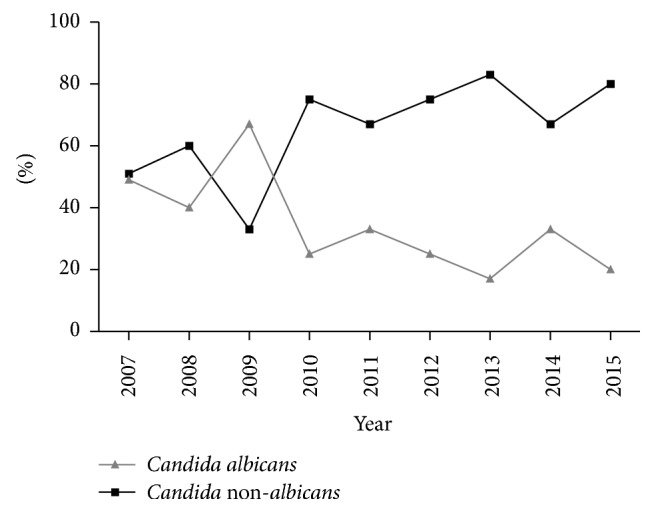
Temporal trend of* Candida albicans* and* Candida *non-*albicans* during a 9-year period.

*Candida parapsilosis sensu stricto* was isolated with the highest frequency (58.5%), followed by* C. albicans* (34.1%),* C. glabrata *complex,* C. guilliermondii,* and* C. orthopsilosis* (2.4% for each). Therefore, 65.9% of candidemia episodes were caused by* Candida *non*-albicans*. With regard to the temporal trend of* C. albicans* and* Candida *non*-albicans*, a variable drift from 2007–2015 was observed, with a considerable percentage (75%) increase in non-*albicans* species in 2015 (Figure 1). Predisposing factors associated with* C. albicans* and non*-albicans* are listed in Table 1. The duration of NICU hospitalization of patients with* C. *non*-albicans* was significantly longer than that in those with* C. albicans* (median days, 10 [7.5–12] versus 12 [10–15], *p* = 0.045). Patients with* C.* non-*albicans* were more likely to have parenteral nutrition than those with* C. albicans* (96.3% versus 71.4%, *p* = 0.039).

Results of antifungal susceptibility are shown in Table 2. All of the strains were sensitive to tested drugs. Overall, the MIC_50_/MIC_90_ values (mg/L) were as follows: amphotericin B, 0.25/0.5; anidulafungin, 1/2; caspofungin, 0.25/0.5; fluconazole, 0.5/2; and micafungin, 1/1.

### 3.2. Literature Review

A total of 45 articles were selected (Tables 3 and 4). Thirty-two studies reported data from a single hospital and 27 were retrospective studies. Seventeen studies were conducted in Asia, 13 in Europe, 11 in North and South America, and 2 in South Africa. Finally, one cohort was carried out in Australia.

**Figure 2:**
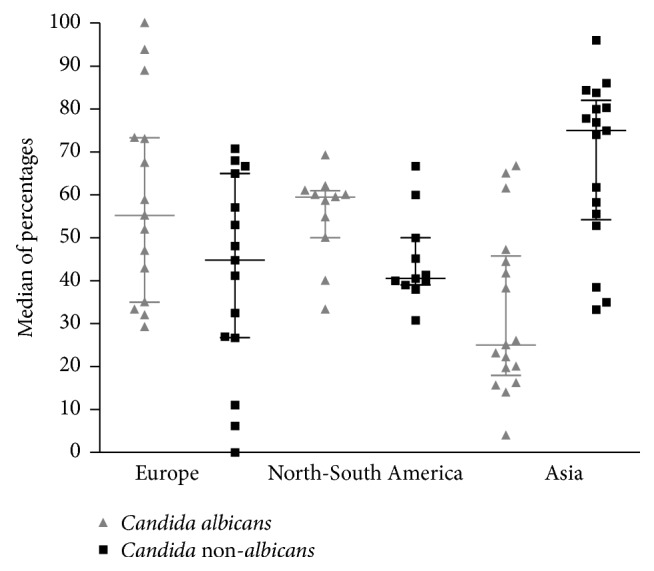
Distribution of* Candid*a spp. according to the different geographical areas.

The distribution of* Candid*a spp. varied according to the different geographical areas.* Candida albicans* was the dominant species in Europe with proportions ranging from 47 to 100% [10, 11, 13, 14, 16, 18, 19, 22, 23, 53] and in North and South America with proportions ranging from 40 to 69.2% [24–31, 33, 34].* Candida* non-*albicans* species were predominant in Asia [36–40, 42, 43, 45, 47, 48], with proportions ranging from 25 to 92%, with a median of 75% (Figure 2). In Australia,* C. albicans* and* C*. non-*albicans* were equally distributed (42% and 43%, resp.) [52].

For* C*. non-*albicans*, the three most prevalent species were* C. parapsilosis* complex,* C. glabrata* complex, and* C. tropicalis*. Generally,* C. parapsilosis* complex was the second most common pathogen (range, 6.2–77.8%).* C. parapsilosis* complex was the predominant species in some studies from Europe [12, 15, 17, 20, 21] and Asia [37, 40, 42, 45, 48]. The highest proportions of* C. glabrata* complex were reported in studies that were conducted in the central part of India (range, 22.2–44.4%), while the lowest proportions were observed in European countries (range, 2.5–5.9%). No cases due to* C. glabrata* complex were reported in South America. The highest frequency of* C. tropicalis* was found in South India (36.7–92%), followed by studies from South America (11.2–13.3%) and South Africa (8.8%). The lowest frequencies were observed in Europe (3.7–5%) and Australia (2%). There were no reports of* C. tropicalis* in North America.

## 4. Discussion

This study aimed to describe the epidemiology and drug susceptibility of* Candida* isolates causing candidemia in a NICU of an Italian university hospital over 9 years. Our survey showed that candidemia is a common problem among critically ill neonates, with an overall incidence of 3%. This finding is higher than data reported in a literature review from Europe (1.1–1.3%) [15, 17] and the North and South America (0.5–1.6%) [25, 30], but lower than that reported in Asia (4–7.7%) [39, 45]. This variability may reflect differences in health care practices among countries, as well as the study design adopted, including differences in the examined population.

VLBW infants are known to be at a high risk of candidemia because of more aggressive and invasive therapies, such as indwelling central lines, mechanical ventilation, parenteral hyperalimentation, and longer hospital stay [1–3]. The majority of infected neonates have a gestational age at birth of 30 weeks or earlier and birth weight is ≤1500 g (87.8%, each one). Intravenous catheters are risk factors for* Candida* BSI in critically ill infants. We found that all patients had intravenous catheter placement and that candidemia was catheter-related in 56.1% of cases. This finding is not surprising because* Candida* spp. can adhere to platelets and fibrinogen on the surface of catheters and form biofilms that may become a reservoir for systemic spread [1–3].

In our systematic review, we found that only four species (*C. albicans*,* C. parapsilosis* complex,* C. tropicalis,* and* C. glabrata* complex) accounted for 95.4% of cases of candidemia. However, the ranking of these four species was variable. Generally,* C. albicans* was the predominant isolated spp. in Europe [10, 11, 13, 14, 16, 18, 19, 22, 23, 53] and North and South America [24–31, 33, 34]. However, non-*albicans* species were predominant in Asia [36–40, 42, 43, 45, 47, 48].

Moreover, data regarding changes in the relative frequencies of isolated* Candida* spp. showed a shift toward* Candida* non-*albicans*, with a frequency higher than 50% in some NICUs. This, in part, is attributed to the increased use of azole prophylaxis and therapy [12]. However, in a recent study, where fluconazole was rarely used for prophylaxis and therapy, a high incidence of non-*albicans* (60.8% of all candidemia episodes) was found [20]. Similarly, our study showed a higher percentage of* C.* non-*albicans* (66%) than* C. albicans* and a variable drift through 9 years. In 2015, 75% of the cases were caused by non-*albicans* species.

In our study, appearance of* C. parapsilosis* complex as the predominant fungal pathogen (61% of all isolates) was consistent with the pattern seen in some hospitals in Europe, Asia, and Africa [12, 15, 17, 20, 37, 42, 45, 48, 51].

Main risk factors for* C. parapsilosis *complex infection were the presence of indwelling vascular catheters and parenteral nutrition, both of which predispose to formation of biofilms. Morphogenesis from yeast cells to pseudohyphae is essential for biofilm formation and virulence in* C. parapsilosis *complex. Amino acids mediate cell differentiation, and this could explain the high incidence of this yeast in catheterized neonates who receive amino acid-rich parenteral nutrition solutions [54]. Our data highlights an association between parenteral nutrition and non*-albicans *spp. The high proportion of* C. parapsilosis *complex may explain this finding. Notably, we observed that NICU patients were more likely to develop* C. parapsilosis sensu stricto* (58.5%) than* C. orthopsilosis* (2.4%) candidemia. This finding may be explained by the greater capacity of* C. parapsilosis sensu stricto* to adhere to central lines compared with closely related species [55].

In agreement with other studies [13–15, 17, 18], none of the isolated strains showed resistance to fluconazole and amphotericin B. These are the antifungal drugs of choice that are used in prophylaxis and treatment of* Candida* BSI in neonates [56]. No fluconazole resistance may be related to the treatment policy in use at our hospital, where systemic antifungal prophylaxis with fluconazole was used only in ELBW infants. In neonates, fluconazole prophylaxis has been linked to the emergence of azole resistance [12, 57].

## 5. Conclusions

Limitations of the present study are mainly related to its retrospective nature with limited follow-up data. Although all of the data were prospectively collected, some variables could not be examined because of missing data. Furthermore, we did not have data on specific characteristics of noninfected patients in our NICU. Therefore, we were not able to risk-adjust our rates to compare with incidences from other reports.

Nevertheless, this study shows that* C. *non*-albicans* candidemia is increasing, despite limited use of fluconazole for prophylaxis/empiric therapy in our unit. Our results also confirm that candidemia plays an important pathogenic role in NICU patients. There is a significant variation in cases of candidemia in different geographic regions, even within the same continent. Therefore, monitoring epidemiological data to facilitate the choice of treatment is important.

## Figures and Tables

**Table 1:** Clinical characteristics of the patients with candidemia by species.

Characteristics	*Candida albicans *(*n* = 14)	*Candida *non-*albicans *(*n* = 27)	*p* value
Low gestational age ≤ 32 wk, *n* (%)	11 (78.6)	25 (92.6)	0.317
Gestational age^‡^	31 (29.5–31.5)	30 (29–31)	0.193
Birth weight ≤ 1500 g, *n* (%)	11 (78.6)	25 (92.6)	0.317
Birth weight (g)^‡^	1200 (1013–1625)	1200 (900–1380)	0.573
Stay in NICU ≤ 7 days, *n* (%)	12 (85.7)	27 (100)	0.111
Length of stay before candidemia (days)^‡^	10 (7.5–12)	12 (10–15)	**0.045**
Presence of CVC, *n* (%)	13 (92.8)	27 (100)	0.342
TPN, *n* (%)	10 (71.4)	26 (96.3)	**0.039**
Mechanical ventilation, *n* (%)	11 (78.6)	26 (96.3)	0.107
Prolonged antibiotic therapy, *n* (%)	12 (85.7)	24 (92.3)	1.000

^‡^Median (interquartile range). CVC: central venous catheter; TPN: total parenteral nutrition. Bold values are significant.

**Table 2:** Cumulative distribution of the MICs of 41 clinical *Candida* isolates.

Isolates (number)	Antifungal drugs	Cumulative % of strains inhibited at the indicated concentrations (mg/L)										
		0.008	0.015	0.03	0.06	0.12	0.25	0.5	1	2	4	8
*Candida parapsilosis *complex (25)	Fluconazole						4	38	77	92	100	
	Amphotericin B				4		58	100				
	Anidulafungin							12	65	100		
	Caspofungin					12	58	100				
	Micafungin							19	92	100		
*Candida albicans *(14)	Fluconazole					17	75	83	100			
	Amphotericin B				8	17	75	100				
	Anidulafungin		33	67	100							
	Caspofungin		12	33	83		100					
	Micafungin	42	100									
All species (41)	Fluconazole					5	25	50	83	93	98	100
	Amphotericin B				5	8	65	100				
	Anidulafungin		10	20	33			40	75	100		
	Caspofungin		5	10	25	33	70	98		100		
	Micafungin	13	30		33			45	93	100		

**Table 3:** Distribution of *Candida* spp. from bloodstream infections in NICU patients from 2000–2015 in various studies.

Reference	Country/observation time	Study design	Number of isolates^a^	Distribution of *Candida* spp. (%)												
				CA	CP	CG	CT	CGU	CF	CK	CL	CD	CLI	CST	CKE	*Candida* spp.^b^
	*Europe*															
Presterl et al., 2007 [10]	Austria/January 2001 to December 2006	Retrospective/single hospital	16	93.8	6.2											
Lagrou et al., 2007 [11]	Belgium/January 2001 to December 2005	Retrospective, data from the hospital information system/single hospital	9	88.9												11.1
Sarvikivi et al., 2005 [12]	Finland/January 2000 to December 2002 (original period: 1991–2002)	Retrospective, data were laboratory-based/single hospital	25	32	68											
Spiliopoulou et al., 2012 [13]	Greece/January 2005 to December 2009	Retrospective/single hospital	40	67.5	25	2.5	5									
Lovero et al., 2016 [14]	Italy/January 2000 to December 2014	Retrospective, data were laboratory-based/single hospital	57	47	44	4		5								
Montagna et al., 2010 [15]	Italy/February 2007 to August 2008	Prospective (Aurora), data were web database-based/6 neonatal units	21	35	60	5										
Tortorano et al., 2013 [16]	Italy/January 2009 to December 2009	Prospective, data were laboratory-based/34 hospitals	17	58.8	35.3	5.9										
Rodriguez et al., 2006 [17]	Spain/January 2002 to December 2003	Prospective, data were laboratory-based/5 hospitals	24	29.2	66.7	4.1										
Pemán et al., 2011 [18]	Spain/January 2009 to February 2010	Prospective (FUNGEMYCA)/30 hospitals	27	51.9	33.3	3.7	3.7	3.7	3.7							
Yalaz et al., 2006 [19]	Turkey/January 2000 to December 2002	Retrospective, review of medical records/single hospital	14	100												
Celebi et al., 2012 [20]	Turkey/January 2000 to December 2007	Prospective/single hospital	28	42.9	57.1											
Ozkan et al., 2014 [21]	Turkey/January 2003 to December 2010	Prospective/single hospital	24	33.3	66.7											
Clerihew et al., 2006 [22]	United Kingdom/February 2003 to February 2004	Prospective (British Paediatric Surveillance Unit)/56 neonatal units	67	55.2	32.8											12
Vergnano et al., 2011 [23]	United Kingdom/January 2006 to December 2008	Prospective (NeonIN), data were web database-based/12 neonatal units	37	73												27

	*North and South America*															
Aziz et al., 2010 [24]	USA/January 2000 to December 2006	Retrospective, review of medical records/single hospital	10	40	40	10							10			
Feja et al., 2005 [25]	USA/March 2001 to January 2003	Prospective/2 neonatal units	45	62	31	2					2			2		
Horn et al., 2009 [26]	USA/July 2004 to March 2008	Prospective (PATH Alliance), data were web database-based/23 hospitals	26	69.2	26.9											3.8
Pfaller et al., 2012 [27]	USA-Canada/July 2004 to December 2008	Prospective (PATH Alliance), data were web database-based/23 medical centers in the USA and two in Canada	62	54.8	30.6	1.6					6.5					6.5
Bizzarro et al., 2015 [28]	USA/January 2004 to December 2013	Retrospective, review of medical records/single hospital	20	50	35	5					5	5				
Natarajan et al., 2009 [29]	USA/January 2006 to December 2007	Retrospective, review of medical records/single hospital	29	58.6	27.6	6.9		3.4		3.4						
Robinson et al., 2012 [30]	USA/January 2000 to December 2010	Retrospective, data from the hospital information system/single hospital	37	59.5	24.3		8.1				5.4					2.7
Batista et al., 2014 [31]	Brazil/October 2006 to March 2007	Prospective/single hospital	10	60	40											
Hoffmann-Santos et al., 2013 [32]	Brazil/January 2006 to December 2011	Retrospective, data were laboratory-based/2 hospitals	45	33.3	48.9		11.2									6.7
Cortés et al., 2011 [33]	Colombia/January 2001 to December 2007	Prospective, data were laboratory-based/27 hospitals	143	61	15				5							19
Cortés et al., 2014 [34]	Colombia/March 2008 to March 2009	Prospective/7 hospitals	15	60	13.3		13.3									13.3

	*Asia*															
Hua et al., 2012 [35]	China/February 2008 to February 2010	Retrospective, review of medical records/single hospital	34	38.2	32.4	2.9	5.9	5.9	11.8	2.9						
Wu et al., 2014 [36]	China/January 2009 to December 2011	Retrospective, review of medical records/single hospital	37	16.2				54.1								29.7
Chen et al., 2015 [37]	China/January 2010 to December 2013	Retrospective, data from the hospital information system/single hospital	43	14	39.5			32.6	14							
Rani et al., 2002 [38]	India/January 2000 to June 2000	Prospective/single hospital	50	4			92								4	
Agarwal et al., 2004 [39]	India/August 2002 to April 2003	Prospective/single hospital	90	15.6												84.4
Femitha et al., 2013 [40]	India/October 2009 and July 2011	Prospective/single hospital	36	25		44.4										30.6
Mehara et al., 2013 [41]	India/January 2012 to September 2012	Retrospective, review of medical records/single hospital	9	44.4		22.2	33.3									
Juyal et al., 2013 [42]	India/January 2012 to December 2012	Prospective, data were laboratory-based/single hospital	132	19.7	25	14.4	24			10.6						8.3
Chaurasia et al., 2015 [43]	India/January 2013 to June 2013	Retrospective, review of medical records/single hospital	30	20	23.3	10	36.7			10						
Wadile and Bhate, 2015 [44]	India/January 2014 to December 2014	Retrospective, review of medical records/single hospital	20	65	15	10	5			5						
Al-Sweih et al., 2009 [45]	Kuwait/January 2000 to December 2006 (original period: 1995–2006)	Retrospective, review of medical records/single hospital	108	41.7	45.4											12.9
Hammoud et al., 2013 [46]	Kuwait/January 2007 to December 2010	Retrospective, review of medical records/single hospital	89	47.2	38.2	6.7		1.1	4.5		2.2					
Khan et al., 2015 [47]	Pakistan/January 2009 to January 2014	Retrospective, data were laboratory-based/single hospital	41	26												74
Wu et al., 2009 [48]	Taiwan/January 2001 to December 2006	Retrospective, review of medical records/single hospital	13	23.1	69.2											7.7
Tsai et al., 2014 [49]	Taiwan/January 2004 to December 2011	Retrospective, review of medical records and administrative databases/single hospital	52	61.5	30.8	7.7										
Lim et al., 2012 [50]	Taiwan/January 2005 to December 2009	Retrospective, review of medical records and administrative database/single hospital	6	66.7	33.3											
Chen et al., 2015 [37]	Taiwan/January 2008 to December 2013	Retrospective, review of medical records/single hospital	9	22.2	77.8											
	*Africa*															
Motara et al., 2005	South Africa/July 2002 to July 2003	Retrospective, data were laboratory-based/single hospital	10	80	20											
Ballot et al., 2013 [51]	South Africa/January 2007 to December 2011	Retrospective/single hospital	57	28.1	56.1	3.5	8.8				1.8	1.8				

	*Oceania*															
Chen et al., 2006 [52]	Australia/August 2001 to July 2004	Retrospective, data were laboratory-based/50 microbiology laboratories	35	42	43	9	2					2				2

CA: *Candida albicans*; CP: *C. parapsilosis*; CG: *C. glabrata*; CT: *C. tropicalis*; CGU: *C. guilliermondii*; CF: *C. famata*; CK: *C. krusei*; CL: *C. lusitaniae*; CD: *C. dubliniensis*; CLI: *C. lipolytica*; CST: *C. stelloidea*; CKE: *C. kefyr*. ^a^Total number of *Candida* isolates from blood (or the total number of candidemia episodes when the number of isolates was not available from the original study). ^b^Including *Candida* spp. not depicted in the table and *Candida* spp. not identified at the species level.

**Table 4:** Main candidemia finding in the NICU as reported in various studies.

Reference	Main candidemia finding in the NICU
Lagrou et al., 2007 [11]	Annual incidence: 0.30 episodes per 10,000 patient-days.
Sarvikivi et al., 2005 [12]	Fluconazole prophylaxis contributed to the emergence of *C. parapsilosis* with decreased susceptibility to fluconazole.
Spiliopoulou et al., 2012 [13]	Candidemia incidence decreased. *C. albicans *was most frequently isolated from ELBW infants. Mortality (35.7%) was associated with low gestational age and low birth weight.
Lovero et al., 2016 [14]	Incidence rate of *Candida* non-*albicans* increased from 46% in 2000–2004 to 71% in 2010–2014.
Montagna et al., 2010 [15]	Overall incidence: 1.3 per 100 NICU discharges. The incidence in ELBW infants was 4.3% versus 0.2% in LBW infants.
Rodriguez et al., 2006 [17]	Annual incidence: 1.1 per 100 NICU discharges and 1.08 per 1000 patient-days. Low mortality (21%) rate may have been caused by a high prevalence of *C. parapsilosis *fungemia.
Pemán et al., 2011 [18]	*C. albicans* was more common in the NICU setting than in the pediatric ICU.
Yalaz et al., 2006 [19]	Candidemia markedly increased in 2002 compared with previous years. A significant association was found between *Candida* infection and the duration of antibiotic therapy.
Celebi et al., 2012 [20]	Overall incidence: 11.5 per 1000 NICU admissions. The mortality rate was 42.8%.
Ozkan et al., 2014 [21]	Gram-positive sepsis (67.6%) was more common than Gram-negative bacteremia (16.6%) and candidemia (15.8%). *Candida* spp. caused LOS (58.3%), VLOS (41,7%), and no EOS sepsis.
Clerihew et al., 2006 [22]	*C. parapsilosis* was associated with fewer deep-seated infections than *C. albicans, *but mortality was similar.
Vergnano et al., 2011 [23]	A decrease in candidemia was observed: 1.8% in 2006, 1.2% in 2007, and 1.3% in 2008. *Candida* spp. were more common in LOS (97%) than in EOS (3%) sepsis.
Aziz et al., 2010 [24]	Fluconazole prophylactic administration to ELBW infants was associated with a decreased rate of candidemia.
Feja et al., 2005 [25]	Overall incidence: 1.6 per 100 NICU discharges. Catheter use, previous bacterial sepsis, and GI pathology were significantly associated with candidemia.
Bizzarro et al., 2015 [28]	*Candida* spp. were more common in LOS than in EOS sepsis.
Natarajan et al., 2009 [29]	Candidemia refractory to conventional antifungals was associated with prolonged antibiotic use and* Candida* non-*albicans* infection.
Robinson et al., 2012 [30]	Overall incidence: 0.45 per 100 NICU discharges. An increased time between blood culture draw and initial antifungal therapy was associated with an increased incidence of persistent candidemia.
Batista et al., 2014 [31]	Oral colonization should be considered as a risk factor for candidemia.
Hua et al., 2012 [35]	Patients with *C. parapsilosis *had a significantly longer hospital stay than those with *C. albicans* sepsis.
Wu et al., 2014 [36]	*C. guilliermondii* was associated with preterm infants and with low birth weight.
Chen et al., 2015 [37]	Fluconazole prophylaxis alone was not efficacious; it had to be combined with reinforcement of management and supervision of hand hygiene to effectively prevent invasive candidiasis.
Rani et al., 2002 [38]	*Candida* non-*albicans* accounted for 96% of the cases of neonatal candidemia.
Agarwal et al., 2004 [39]	Overall incidence: 77 per 1000 NICU discharges. *Candida* non-*albicans* is gaining importance as a cause of neonatal septicemia.
Femitha et al., 2013 [40]	Overall incidence: 0.82 cases per 100 NICU discharges. Mortality was 44.4%. Presence of candiduria was a significant riskfactor for death.
Mehara et al., 2013 [41]	*Candida* spp. were more common in LOS than in EOS sepsis.
Juyal et al., 2013 [42]	*Candida *non-*albicans *accounted for 80.30% of the cases of neonatal candidemia. The crude mortality was 34.85%.
Chaurasia et al., 2015 [43]	Clinical features in neonates with candida sepsis were nonspecific. A common laboratory feature was thrombocytopenia.
Al-Sweih et al., 2009 [45]	Overall incidence: 4 per 100 NICU discharges.
Hammoud et al., 2013 [46]	*C. albicans* was the most prevalent species in nonpersistent candidemia. *C. parapsilosis* was more common among infants with persistent candidemia. Persistent candidemia was associated with an increased risk of mortality.
Wu et al., 2009 [48]	The most common causative microorganisms of LOS sepsis were CONS and *Candida* spp. *C. parapsilosis* was associated with a high mortality rate.
Tsai et al., 2014 [49]	Candidemia had a significantly higher rate of infectious complications, persistent bloodstream infection, and sepsis-attributable mortality than Gram-negative and Gram-positive bacteremia.
Lim et al., 2012 [50]	Sepsis by Gram-negative bacteria or *Candida* spp. presented with more severe clinical symptoms and was associated with a higher mortality rate compared with that by Gram-positive bacteria.
Chen et al., 2015 [37]	Decrease incidence of candidemia during the study period.
Ballot et al., 2013 [51]	Increased incidence of *Candida* non-*albicans *during the study period.

CONS: coagulase-negative staphylococci; ELBW: extremely low birth weight; VLBW: very low birth weight; GI: gastrointestinal; EOS: early-onset sepsis; LOS: late-onset sepsis; VLOS: very late-onset sepsis; NICU: neonatal intensive care unit; ICU: intensive care unit.
